# Foodborne and Waterborne Infections in Elderly Community and Long-Term Care Facility Residents, Victoria, Australia

**DOI:** 10.3201/eid1803.110311

**Published:** 2012-03

**Authors:** Martyn D. Kirk, Joy Gregory, Karin Lalor, Gillian V. Hall, Niels Becker

**Affiliations:** Australian National University, Canberra, Australian Capital Territory, Australia (M.D. Kirk, G.V. Hall, N. Becker);; Department of Health, Melbourne, Victoria, Australia (J. Gregory, K. Lalor)

**Keywords:** foodborne, waterborne, enteric infections, long-term care facility, incidence, elderly, bacteria, Australia

## Abstract

We calculated rates of foodborne and waterborne infections reported to the health department in Victoria, Australia, during 2000–2009 for elderly residents of long-term care facilities (LTCFs) and the community. We used negative binomial regression to estimate incidence rate ratios, adjusting for age, sex, and reporting period. We analyzed 8,277 infections in elderly persons. Rates of campylobacteriosis, legionellosis, listeriosis, toxigenic *Escherichia coli* infections, and shigellosis were higher in community residents, and rates of *Salmonella* infection were higher in LTCF residents. Each year, 61.7 *Campylobacter* infections were reported per 100,000 LTCF residents, compared with 97.6 per 100,000 community residents. LTCF residents were at higher risk for *S. enterica* serotype Typhimurium associated with outbreaks. Rates of foodborne infections (except salmonellosis) were similar to or lower for LTCF residents than for community residents. These findings may indicate that food preparation practices in LTCFs are safer than those used by elderly persons in the community.

## Abstract 2

LTCF residents had lower or similar rates of these infections, except salmonellosis, than community residents.

Infectious disease incidence varies with age, and elderly persons are considered more vulnerable than younger persons to foodborne and waterborne infections (*1**,**2*). In many countries, elderly persons unable to care for themselves live in long-term care facilities (LTCFs) where they receive assistance with meals, daily living, and health care (*3*). Food preparation practices and various exposures in LTCFs may modify the risk of foodborne and waterborne infections in these residents (*4**,**5*) when compared with elderly persons living in the community, who may have less safe food preparation practices (*6**–**8*).

A variety of pathogens transmitted by food or water, including *Campylobacter* sp., *Clostridium perfringens*, *Cryptosporidium* sp., *Legionella* spp., and *Shigella* sp., and various serotypes of *Salmonella enterica* can infect humans (*9**,**10*). Foodborne and waterborne infections predominantly manifest in elderly persons as gastroenteritis but, depending on the infectious agent, can result in pneumonia, bacteremia, and meningitis (*11**,**12*). Elderly persons can become infected by ingesting contaminated water or food or, as with *Legionella* spp., inhaling contaminated aerosols (*13*). Some infections are predominantly foodborne; others can be acquired from infected persons or animals or through contact with contaminated environments (*4*).

These agents can manifest as outbreaks in facilities, leading to community concern about the safety of residents (*14**,**15*). Although most outbreaks of gastroenteritis in LTCFs are spread from person to person and are generally mild (*16*), such outbreaks do result in higher case-fatality rates (CFRs) among residents (*17*). As a result, regulatory agencies in many countries have mandated programs to manage food safety in facilities. To prevent legionellosis in residents, health agencies commonly provide advice about disinfection of hot water systems that can be reservoirs for *Legionella* spp (*13*).

Few studies have compared the incidence of infections caused by agents that can be transmitted by contaminated food or water consumed by elderly persons living in LTCFs and in the community. One study in the United States estimated that the lower limit of the death rate for nursing home residents from gastroenteritis of unknown etiology was 38.91 (95% CI 38.55–39.27) per 100,000 persons per year, compared with an estimated upper limit of 8.50 (95% CI 8.47–8.53) per 100,000 persons >65 years of age living in the community (*18*). Little examination has been done of the incidence of sporadic foodborne or waterborne diseases in institutionalized elderly persons. To address this gap, we estimated rates of reported infection in persons >65 years of age living in Victoria, Australia, infected with any of 7 different pathogens according to whether they lived in a government-subsidized LTCF or in the community, and we examined the effect of age on incidence of disease. These pathogens were *Campylobacter* sp., *Cryptosporidium* sp., *Legionella* spp., *Listeria* sp., *Salmonella enterica*, Shiga toxin–producing *Escherichia coli* (STEC), and *Shigella* sp.

## Methods

### Infectious Disease Surveillance

Victoria is 1 of 6 states and 2 territories in Australia. Public health legislation mandates that all physicians and pathology laboratories in Victoria report cases of notifiable conditions under the Public Health and Wellbeing Regulations 2009 (www.health.vic.gov.au/ideas/notifying/whatto) to the state’s Department of Health. Health department staff members enter details about reported cases into a database. Among the 64 conditions notifiable as of April 14, 2010, a total of 14 were enteric diseases can be transmitted by contaminated food or water and 1 was *Legionella* infection that could potentially be transmitted by inhalation of contaminated water. Surveillance for these diseases has remained essentially unchanged in Victoria since the early 1990s, except for cryptosporidiosis, for which reporting was voluntary until 2001, when notification became mandatory by law.

We analyzed data on all cases of campylobacteriosis, cryptosporidiosis, legionellosis, listeriosis, shigellosis, salmonellosis, and STEC infection that were reported to Victoria’s Department of Health during January 1, 2000–December 31, 2009. Surveillance officers recorded whether cases were part of an outbreak or occurred in persons who had traveled overseas during their incubation period, which varied for different diseases. In addition, to identify information that was not recorded by surveillance officers, we reviewed surveillance data for all LTCF residents. More specifically, we identified where >2 cases of the same pathogen occurred within the same facility within 2 weeks, and we recoded these cases as outbreak associated. Surveillance officers also recorded whether case-patients had died of the disease or of other concurrent conditions within the weeks after infection during public health follow-up.

### Data Analysis

We categorized reported cases by residential status of the patient. An LTCF resident was a person who had a residential address of a government-subsidized LTCF, and a community resident was a person living in a private residence in Victoria (Technical Appendix). We excluded from analysis cases in persons without a valid address because we assumed the address was missing completely at random. We also excluded case-patients residing in privately funded facilities (supported residential services) catering to elderly or disabled persons or persons with psychiatric illness or dementia because they were not included in the denominator of LTCF residents. We counted the annual number of foodborne and waterborne infections, including those from epidemiologically important serotypes and species, in LTCF and community residents during the 10-year period. We obtained age-specific annual denominator data for residents of government-subsidized LTCFs from annual reports prepared by the Australian Institute of Health and Welfare (*19*). Denominator data for community residents were calculated by subtracting annual age-specific estimates of LTCF residents from estimated resident populations prepared by the Australian Bureau of Statistics (www.abs.gov.au/). We calculated annual rates of notification for different diseases by residential status and compared them with the total rate of notifications for the state. We calculated CFRs by dividing the number of deaths from the disease in the different groups by the total number of cases of disease, including cases for which death status was unknown, for the 10-year period. To account for age differences in LTCF and community residents, we calculated age-adjusted relative risks (RRs) for death from infection with different pathogens by using Mantel-Haenszel methods.

To estimate incidence rate ratios (IRRs), we used a negative binomial regression model of the annual count of foodborne and waterborne infections by the period of notification (2000–2004 and 2005–2009), sex, 3 categories of age (65–74, 75–84, and >85 years), and residence (LTCF and community). The number of persons living in LTCFs or the community in each age group for each year was entered into the model as an offset. We used robust variance estimation suited to longitudinal or clustered data to account for possible clustering from outbreaks. We assessed model fit by examining the distribution of standardized Pearson residuals. To assess the effect on incidence, we repeated regression models excluding travel-associated cases and including only the first case for each known outbreak in LTCFs and the community. We analyzed data by using Stata version 11.2 (Stata Corp., College Station, TX, USA).

The Australian National University human research ethics committee approved this study. Victoria’s Department of Health approved release of the data.

## Results

### Incidence in Persons >65 Years of Age

During January 1, 2000–December 31, 2009, a total of 8,534 cases of the 7 diseases were reported in persons >65 years of age. During data cleaning, we excluded 238 (2.8%) persons without valid residential addresses and 19 who lived in a private institution caring for elderly or disabled persons. A total of 8,277 cases were available for analysis, including 132 (1.6%) case-patients living in retirement villages.

### Infections in Institutional and Community Residents

**Figure 1:**
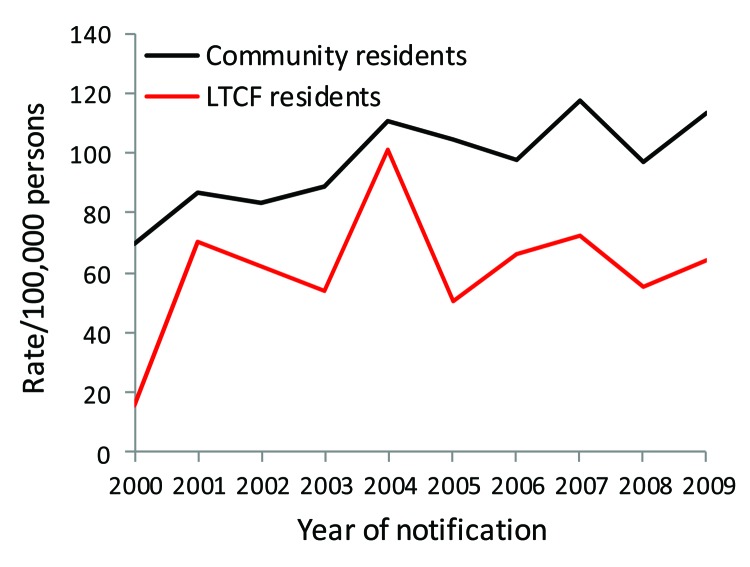
Notification rates for campylobacteriosis in persons >65 years of age, by long-term care facility (LTCF) and community residence status, Victoria, Australia, 2000–2009.

**Figure 2:**
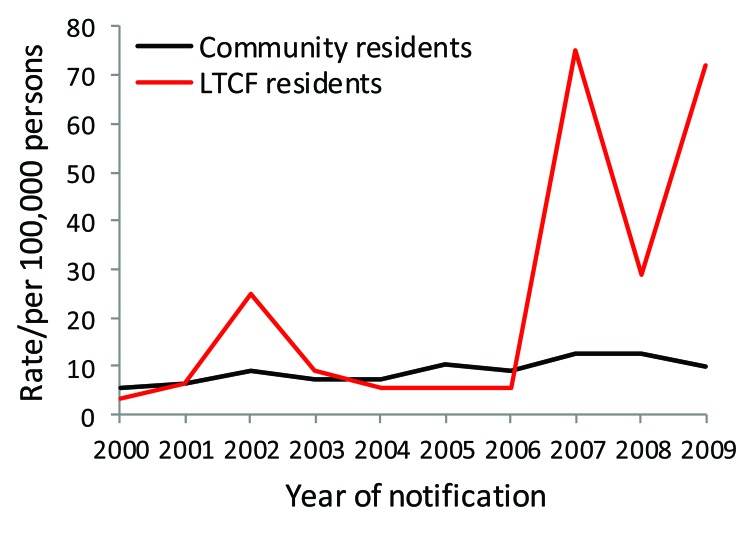
Notification rates for *Salmonella enterica* serotype Typhimurium infections in persons >65 years of age, by long-term care facility (LTCF) and community residence status, Victoria, Australia, 2000–2009.

Rates of all reported infections in LTCF residents were similar to or lower than those in community residents, except infections from *S. enterica* (Table 1). No LTCF residents were reported with *L. longbeachae*, STEC, or *Shigella* sp. infections during the surveillance period. Among persons >65 years of age infected with *Legionella* species other than *L. longbeachae* were more likely to live in an LTCF (crude RR 1.15, 95% CI 1.1–1.2; p = 0.27). The reported rate of *Campylobacter* spp. was lower in LTCF residents than in community residents for all years of surveillance (Figure 1). In contrast, reports of *S. enterica* serotype Typhimurium peaked because of outbreaks during the study period, which resulted in higher rates for LTCF residents overall (Figure 2).

**Table 1:** Incidence rate for reported infections with pathogens possibly transmitted by food or water, Victoria, Australia, January 2000–December 2009*

Pathogen	Persons <65 y			Persons >65 y							Total reports	
	No. cases	Rate		LTCF residents			Community residents		Missing address/ excluded facility		No. cases	Rate
				No. cases	Rate		No. cases	Rate				
*Campylobacter* sp.	50,444	115.4		215	61.7		6,207	97.6	206		57,072	113.2
*Cryptosporidium* sp.	4,955	11.3		7	2.0		106	1.7	3		5,071	10.1
*Legionella pneumophila*/ other	457	1.0		8	2.3		293	4.6	4		762	1.5
*L. longbeacheae*	49	0.1		0	0.0		45	0.7	0		94	0.2
*Listeria monocytogenes†*	46	0.1		3	0.9		70	1.1	4		123	0.2
*Salmonella enterica* serotype Typhimurium	7,204	16.5		87	25.0		585	9.2	19		7,895	15.7
*S. enterica,* other serotypes	5,003	11.4		44	12.6		552	8.7	20		5,619	11.1
Shiga toxin–producing *Escherichia coli*	56	0.1		0	0.0		12	0.2	1		69	0.1
*Shigella* sp.	845	1.9		0	0.0		43	0.7	0		888	1.8

*Annual rate of reported infections per 100,000 persons. LTCF, long-term care facility. †Total listeriosis reports exclude 16 pregnancy-associated infections**.**

### Associated Death

The CFR was highest for listeriosis and *L. longbeachae* infection, from which 17.8% and 6.7% of persons died, respectively (Table 2). The age-adjusted RR for death from *L. monocytogenes* infections was 3.5 (95% CI 1.2–10.4; p = 0.03) for LTCF residents. No *L. pneumophila*–associated deaths were recorded in LTCF residents. For salmonellosis, most deaths were considered to have resulted from other concurrent conditions. The age-adjusted RR for death in LTCF residents infected with any *S. enterica* serotype did not differ significantly from that in community residents (adjusted RR 2.8, 95% CI 0.8–9.1; p = 0.87). Death status was not routinely ascertained for persons with campylobacteriosis, although 1 death from campylobacteriosis and 11 deaths from concurrent conditions were recorded. No deaths were recorded for shigellosis or cryptosporidiosis.

**Table 2:** Deaths associated with infections from foodborne and waterborne pathogens or concurrent conditions reported in residents >65 years of age in long-term care facilities and the community, Victoria, Australia, January 2000–December 2009

Pathogen	Died of disease	Died of concurrent condition	Death status unknown	No. cases	Case-fatality rate*
*Legionella pneumophila*/other	11	1	169	301	4.0
*L. longbeacheae*	2	1	0	45	6.7
*Listeria monocytogenes*	4	9	14	73	17.8
*Salmonella enterica* serotype Typhimurium	3	8	183	672	1.6
*S. enterica*, other serotypes	0	7	177	596	1.2
Shiga toxin–producing *Escherichia coli*	1	0	1	12	8.3
*Shigella* sp.	0	0	10	43	NA

*Per 100 cases. Case-fatality rate for the 10-year study period was calculated by dividing the total number of deaths among case-patients by all case-patients, including those with unknown death status. NA, not applicable.

### Rates of Foodborne and Waterborne Infections

In multivariable analysis, the incidence of *S. enterica* serotype Typhimurium was higher in LTCF residents than in community residents (IRR 2.3, 95% CI 1.6–3.4; p<0.001) and non-Typhimurium serotypes of *S. enterica* (IRR 1.4, 95% CI 1.0–1.9; p = 0.04) and lower for *Campylobacter* spp. (IRR 0.63, 95% CI 0.52–0.77; p<0.001) (Table 3). IRRs could not be estimated for *L. longbeachae* and STEC infections and for shigellosis because no cases occurred in LTCF residents during the surveillance period. We observed a trend of increasing rates of reported infections for cryptosporidiosis, salmonellosis, and campylobacteriosis over time during the surveillance period.

**Table 3:** Adjusted incidence rate ratios from multivariable model for foodborne and waterborne infections reported in residents >65 years of age in long-term care facilities and the community, Victoria, Australia, January 2000–December 2009

Variable	Incidence rate ratio (95% CI)					
	*Cryptosporidium*,* n = 113	*Listeria monocytogenes*, n = 73	*Salmonella enterica* serovar Typhimurium, n = 662	*S. enterica*, other serotypes, n = 586	*L. pneumophila*/ other, n = 301	*Campylobacter* spp., n = 6,387
Sex						
F	1.0	1.0	1.0	1.0	1.0	1.0
M	0.86 (0.51–1.4)	1.5 (0.90–2.6)	0.97 (0.80–1.2)	0.97 (0.80–1.2)	2.6 (1.7–4.1)	1.2 (1.1–1.3)
Year						
2000–2004	1.0	1.0	1.0	1.0	1.0	1.0
2005–2009	3.7 (2.1–6.6)	1.2 (0.68–1.9)	1.8 (1.4–2.2)	1.7 (1.4–2.1)	0.35 (0.23–0.53)	1.2 (1.1–1.3)
Age group, y						
65–74	1.0	1.0	1.0	1.0	1.0	1.0
75–84	0.92 (0.53–1.6)	2.0 (1.1–3.5)	1.1 (0.95–1.4)	1.1 (0.86–1.3)	1.5 (0.90–2.5)	1.1 (1.0–1.2)
>85	0.58 (0.31–1.1)	2.8 (1.2–6.4)	1.3 (0.99–1.8)	1.1 (0.80–1.4)	1.2 (0.63–2.2)	1.1 (1.0–1.2)
Long-term care facility resident						
No	1.0	1.0	1.0	1.0	1.0	1.0
Yes	1.4 (0.74–2.8)	0.56 (0.10–3.0)	2.3 (1.6–3.4)	1.4 (1.0–1.9)	0.57 (0.27–1.2)	0.63 (0.52–0.77)

*In Victoria, reporting of cryptosporidiosis was voluntary until 2001, when notification became mandatory by law.

From multivariable analysis, reported incidence rates were higher in older age groups for *L. monocytogenes, Campylobacter* sp., and *S. enterica* serotype Typhimurium infections than in the base age group of persons 65–74 years (Table 3). The incidence of *L. pneumophila* and non-Typhimurium serotypes of *S. enterica* infections and cryptosporidiosis did not differ significantly by age group.

### Accounting for Travel and Outbreaks

During their incubation period, 105 (1.3%) of the 8,277 persons with notifiable infections traveled internationally; all were community residents. Among community residents, 16 (37.2%) of the 43 with shigellosis and 48 (8.7%) of the 552 with non-Typhimurium serotypes of *S. enterica* had traveled internationally, compared with 0 of those with *L. monocytogenes*, *L. longbeachae*, and STEC infections. Only 35 (0.6%) of the 6,207 community residents infected with *Campylobacter* sp. were recorded as traveling overseas before infection.

During the study period, 42 separate outbreaks of *S. enterica* serotype Typhimurium occurred in persons >65 years of age. There were also 36 outbreaks of non-Typhimurium serotypes of *S. enterica*, 14 of *L. pneumophila*/other, 10 of *Campylobacter* spp. infections, and 2 of *Shigella* sp*.* In total, 189 (2.4%) of 7,913 cases in community residents were recorded as outbreak associated. In LTCF residents, 111 (30.5%) of the 364 cases were outbreak associated, including 68 (78.2%) of the 87 *S. enterica* serotype Typhimurium and 33 (15.3%) of the 215 *Campylobacter* spp. infections. No cases of cryptosporidiosis, listeriosis, or STEC infection were recorded as outbreak associated in either LTCF residents or community residents.

When we repeated multivariable models, excluding travel-associated infections and including a single case for each identified outbreak, the incidence rate for *S. enterica* serotype Typhimurium was similar in LTCF residents and community residents (IRR 0.91, 95% CI 0.64–1.29; p = 0.59). For infections with non-Typhimurium serotypes of *S. enterica*, the incidence was higher in LTCF residents than in community residents (IRR 1.4, 95% CI 1.0–2.0; p = 0.05). After adjustment for travel and outbreaks, the incidence rate was lower for LTCF residents with *Campylobacter* spp. (IRR 0.57, 95% CI 0.48–0.68; p<0.001). For *L. pneumophila*, the incidence rate was lower, but not significantly, for LTCF residents (IRR 0.63, 95% CI 0.29–1.3; p = 0.23).

## Discussion

Rates of foodborne and waterborne infections among LTCF residents were lower than or similar to rates among community residents, except for salmonellosis, which was higher. In particular, rates of campylobacteriosis in LTCF residents were consistently lower throughout the entire study period, which was unexpected because incidence of this infection is universally high (*20*). Despite the high incidence of campylobacteriosis, outbreaks are rare in Australia, possibly because of the high dose required to cause infection and because foods causing infection are thought to become contaminated through cross-contamination (*16**,**20*). In Australia, *Campylobacter* infections are the most common cause of bacterial foodborne disease, with contaminated chicken meat causing ≈30% of all infections each year (*21**–**23*).

The lower incidence of campylobacteriosis might result from the highly regulated food hygiene system for LTCFs. In 1998, Victoria was the first Australian state to implement mandatory food safety programs for food service settings, and those serving vulnerable populations require independent auditing (*24*). These programs might have resulted in better understanding and practices by LTCF staff about food storage, cooking, and cross-contamination than by elderly persons in their own homes. *Campylobacter* infections in elderly community residents have been associated with risk possibly from cross-contamination during food preparation (*25*). Even though *Campylobacter* infections are associated with travel, a case–control study in Australia found that only 23 (2.8%) of 833 persons >5 years of age with campylobacteriosis had traveled overseas during the week before illness (*21*). When we accounted for known travel history and outbreak-associated cases, the IRR for *Campylobacter* spp. infections in LTCF residents was lowered.

*S. enterica* is a common cause of foodborne and waterborne outbreaks in LTCFs (*17**,**26**,**27*), a finding that our study confirmed. We found that outbreaks of *S. enterica* serotype Typhimurium infections accounted for the higher incidence of these infections, but not for non-Typhimurium serotype infections, in LTCF residents. Sources of outbreaks in LTCFs often are not identified, although eggs are commonly suspected as the cause in *S. enterica* serotype Typhimurium–associated outbreaks (*17*). Although residents are at higher risk for outbreak-associated disease, ascertainment of cases is biased in the institutional setting because of the common living environment, centralized access to health care, and collection of specimens by public health staff. During outbreaks, public health investigators often collect fecal specimens from LTCF residents with diarrhea, which would not occur for elderly persons in the community. Because surveillance is well established in Victoria, LTCFs are more likely than persons in the community to report outbreaks (*16*).

We did not find any evidence to suggest that living in an LTCF increased a person’s risk for legionellosis, despite the occasional occurrence of outbreaks and sporadic cases in this setting (*13**,**28*). LTCF residents reported with legionellosis were more likely to be infected with *Legionella* species other than with *L. longbeachae*. *L. longbeachae* is associated with gardening and potting mix, so we expected the incidence of this infection to be low in LTCF residents (*29*).The incidence of listeriosis was similar in LTCF and community residents. Given the food safety program requirements in facilities in Victoria, LTCF residents plausibly could be exposed to lower concentrations of *L. monocytogenes* in food, compared with community residents who may keep food longer, have poorer food preparation practices, and eat foods considered higher risk for transmitting foodborne pathogens (*8*).

Different clinical investigative approaches for LTCF and community residents with potential foodborne and waterborne disease might account for some of our findings. Although clinicians might elect not to collect specimens when LTCF residents have diarrheal illness, we think it more likely that reporting is more complete in LTCFs. Most of the diseases in our study are serious illnesses, and infected persons would have severe gastrointestinal and extraintestinal symptoms lasting for several days or weeks (*3**,**4*). In a case–control study of campylobacteriosis in Australia, 41% of case-patients had bloody diarrhea, and 75% had fever; both of these symptoms are strong predictors for physicians ordering laboratory tests (*30**,**31*).

We were unable to control for potential confounding factors, such as concurrent conditions and factors that might predispose for infection. Many elderly persons with concurrent conditions live in the community, but the health status of LTCF residents is likely to be lower, and they are likely to be more frail. We would have partly controlled for frailty through inclusion of age in our multivariable model because elderly persons in institutions are the oldest and the most frail in society (*32*). In addition, our study was underpowered to detect an effect for diseases where notification rates were very low. The potential bias in the final estimates from lack of control of confounding would be more likely to result in increased incidence rates in LTCF residents. However, except for salmonellosis, infection rates were higher in community residents.

Our findings should not be overinterpreted because our study was a retrospective record–based study in which we manually coded surveillance data and were unable to validate case-patients’ addresses. It is possible that we were unable to correctly identify residential status of case patients from addresses. In some instances, residents were recorded as living at addresses where an LTCF and retirement village were on the same grounds, making determining whether a person lived in the facility difficult. Similarly, some persons might have been infected after moving into an LTCF, but the address on a pathology report still recorded their residential address in the community where they had previously lived. However, in Victoria, physicians and laboratories were required to report these infections, making it unlikely that both sources of notification would incorrectly report the residential address. For *Campylobacter* infections, however, physicians report only 50% of notifications, with the remainder coming from laboratories (*33*).

Elderly community residents might receive meals from organizations that provide community support. In addition, elderly residents of LTCF might eat food that has been prepared outside the facility during excursions or brought in by visitors, which could result in exposure to foodborne pathogens. For both groups, these alternative routes of exposure would modify the risk for infection so that it did not truly reflect the risk in their place of residence.

The strength of our approach was that we consistently coded addresses without regard to disease-causing agent, yet we observed distinct differences in reported incidence from disease to disease. Our findings were consistent with what we know about these diseases, such as increasing incidence in older persons for diseases such as listeriosis. The CFRs were consistent with reports in the literature for elderly persons, although we assessed deaths only short term (i.e., in the weeks after infection) (*34**,**35*). In general, elderly persons have more severe outcomes from foodborne infections than do younger persons (*4**,**18*). Large-scale studies that used population-based registers have demonstrated that enteric diseases contribute to more deaths than recognized from short-term follow-up, even when controlling for concurrent conditions (*36**,**37*).

In our study, rates of surveillance reports for most infections in persons >65 years of age were similar to or lower than for persons <65 years of age, a finding that contradicts the common statement that elderly persons are at higher risk for foodborne disease. However, we did find that the CFRs were high for some infections and that LTCF residents were affected more severely. We believe that our findings can be generalized to other Australian states and territories with similar rates of infection and methods of surveillance (*16**,**22*). Other investigators could repeat this study by using record-linkage to compare their findings with our findings.

We observed a lower incidence of reported *Campylobacter* spp. infection in LTCF residents, which provides some reassurance for food safety regulators and the aged care industry. Our study highlights that most foodborne and waterborne infections are rare in elderly residents of LTCF and the community, but that these infections do cause occasional deaths. Primary research is needed into the specific causes of foodborne and waterborne infections in elderly persons in the community and in institutional settings that particularly accounts for the effect of concurrent conditions. In our study, elderly LTCF residents had an incidence of foodborne and waterborne infections that was similar to or lower than that that for elderly persons living in the community, except for *S. enterica* infections.

## Supplementary Material

Technical Appendix Coding Surveillance Data for Residential Status Surveillance in Victoria, Australia.

## References

[1] Smith JL. Foodborne illness in the elderly. J Food Prot. 1998;61:1229–39. 9766083 10.4315/0362-028x-61.9.1229

[2] Lund BM, O'Brien SJ. The occurrence and prevention of foodborne disease in vulnerable people. Foodborne Pathog Dis. 2011;8:961–73. 10.1089/fpd.2011.0860 21561383 PMC3159107

[3] Smith PW, Bennett G, Bradley S, Drinka P, Lautenbach E, Marx J, SHEA/APIC guideline: infection prevention and control in the long-term care facility, July 2008. Infect Control Hosp Epidemiol. 2008;29:785–814. 10.1086/592416 18767983 PMC3319407

[4] Kirk MD, Veitch MG, Hall GV. Gastroenteritis and foodborne disease in elderly people living in long-term care. Clin Infect Dis. 2010;50:397–404. 10.1086/649878 20047497

[5] Gavazzi G. Ageing and infection. Lancet Infect Dis. 2002;2:659–66. 10.1016/S1473-3099(02)00437-1 12409046

[6] Nesbitt A, Majowicz S, Finley R, Marshall B, Pollari F, Sargeant J, High-risk food consumption and food safety practices in a Canadian community. J Food Prot. 2009;72:2575–86. 20003742 10.4315/0362-028x-72.12.2575

[7] Sellers T, Andress E, Fischer JG, Johnson MA. Home food safety program for the Georgia Older Americans Act Nutrition Program. J Nutr Elder. 2006;26:103–22. 10.1300/J052v26n01_06 17890206

[8] Johnson AE, Donkin AJ, Morgan K, Lilley JM, Neale RJ, Page RM, Food safety knowledge and practice among elderly people living at home. J Epidemiol Community Health. 1998;52:745–8. 10.1136/jech.52.11.745 10396508 PMC1756646

[9] Tauxe RV, Doyle MP, Kuchenmuller T, Schlundt J, Stein CE. Evolving public health approaches to the global challenge of foodborne infections. Int J Food Microbiol. 2010;139(Suppl 1):S16–28. 10.1016/j.ijfoodmicro.2009.10.014 19931203

[10] Hall G, Kirk MD, Becker N, Gregory JE, Unicomb L, Millard G, Estimating foodborne gastroenteritis, Australia. Emerg Infect Dis. 2005;11:1257–64. 16102316 10.3201/eid1108.041367 PMC3320479

[11] Beheshti M, George WL. Infectious diarrhea. In: Yoshikawa T, Norman DC, editors. Infectious disease in the aging: a clinical handbook. 2nd ed. New York: Humana Press; 2009. p.143–63.

[12] Cates SC, Kosa KM, Moore CM, Jaykus LA, Ten Eyck TA, Cowen P. Listeriosis prevention for older adults: effective messages and delivery methods. Educ Gerontol. 2007;33:587–606. 10.1080/03601270701411023

[13] Seenivasan MH, Yu VL, Muder RR. Legionnaires' disease in long-term care facilities: overview and proposed solutions. J Am Geriatr Soc. 2005;53:875–80. 10.1111/j.1532-5415.2005.53270.x 15877568

[14] Kirk MD, Roberts L, Horvath J. Understanding gastroenteritis in elderly Australians. Med J Aust. 2008;189:476–7. 18976184 10.5694/j.1326-5377.2008.tb02136.x

[15] Trop Skaza A, Beskovnik L, Storman A, Ursic S, Groboljsek B, Kese D. Outbreak of Legionnaires' disease in a nursing home, Slovenia, August 2010: preliminary report. Euro Surveill. 2010;15:19672. 20929657

[16] Kirk MD, Fullerton K, Hall GV, Gregory J, Stafford R, Veitch MG, Surveillance for outbreaks of gastroenteritis in long-term care facilities, Australia, 2002–8. Clin Infect Dis. 2010;51:907–14. 10.1086/656406 20825308

[17] Kirk MD, Lalor K, Raupach J, Combs B, Stafford R, Hall GV, Food- and waterborne disease outbreaks in Australian long-term care facilities, 2001–2008. Foodborne Pathog Dis. 2011;8:133–9. 10.1089/fpd.2010.0648 21034268

[18] Frenzen PD. Mortality due to gastroenteritis of unknown etiology in the United States. J Infect Dis. 2003;187:441–52. 10.1086/368097 12552428

[19] Australian Institute of Health and Welfare. Residential aged care in Australia 2007–08: a statistical overview.[cited 2010 Aug 4]. http://www.aihw.gov.au/publication-detail/?id=6442468253&tab=2

[20] Monitoring the incidence and causes of diseases potentially transmitted by food in Australia: annual report of the OzFoodNet Network, 2008. Commun Dis Intell. 2009;33:389–413. 20301968 10.33321/cdi.2009.33.42

[21] Stafford RJ, Schluter P, Kirk M, Wilson A, Unicomb L, Ashbolt R, A multi-centre prospective case-control study of campylobacter infection in persons aged 5 years and older in Australia. Epidemiol Infect. 2007;135:978–88. 10.1017/S0950268806007576 17134530 PMC2870644

[22] Kirk MD, McKay I, Hall GV, Dalton CB, Stafford R, Unicomb L, Food safety: foodborne disease in Australia: the OzFoodNet experience. Clin Infect Dis. 2008;47:392–400. 10.1086/589861 18558879

[23] Stafford RJ, Schluter PJ, Wilson AJ, Kirk MD, Hall G, Unicomb L. Population-attributable risk estimates for risk factors associated with *Campylobacter* infection, australia. Emerg Infect Dis. 2008;14:895–901. 10.3201/eid1406.071008 18507899 PMC2600281

[24] Victorian Competition and Efficiency Commission. Simplifying the menu: food regulation in Victoria [cited 2010 Aug 4]. http://www.vcec.vic.gov.au/CA256EAF001C7B21/WebObj/FoodRegulationDraftReport/$File/Food%20Regulation%20Draft%20Report.pdf

[25] Doorduyn Y, Van Den Brandhof WE, Van Duynhoven YT, Breukink BJ, Wagenaar JA, Van Pelt W. Risk factors for indigenous *Campylobacter jejuni* and *Campylobacter coli* infections in the Netherlands: a case-control study. Epidemiol Infect. 2010;138:1391–404. 10.1017/S095026881000052X 20223048

[26] Levine WC, Smart JF, Archer DL, Bean NH, Tauxe RV. Foodborne disease outbreaks in nursing homes, 1975 through 1987. JAMA. 1991;266:2105–9. 10.1001/jama.1991.03470150077034 1656108

[27] Ryan MJ, Wall PG, Adak GK, Evans HS, Cowden JM. Outbreaks of infectious intestinal disease in residential institutions in England and Wales 1992–1994. J Infect. 1997;34:49–54. 10.1016/S0163-4453(97)80009-6 9120324

[28] Polverino E, Dambrava P, Cilloniz C, Balasso V, Marcos MA, Esquinas C, Nursing home-acquired pneumonia: a 10 year single-centre experience. Thorax. 2010;65:354–9. 10.1136/thx.2009.124776 20388763

[29] O'Connor BA, Carman J, Eckert K, Tucker G, Givney R, Cameron S. Does using potting mix make you sick? Results from a *Legionella longbeachae* case–control study in South Australia. Epidemiol Infect. 2007;135:34–9. 10.1017/S095026880600656X 16780608 PMC2870547

[30] Unicomb LE, Fullerton KE, Kirk MD, Stafford RJ. Outbreaks of campylobacteriosis in Australia, 2001 to 2006. Foodborne Pathog Dis. 2009;6:1241–50. 10.1089/fpd.2009.0300 19895264

[31] Hall G, Yohannes K, Raupach J, Becker N, Kirk M. Estimating community incidence of *Salmonella, Campylobacter*, and Shiga toxin–producing *Escherichia coli* infections, Australia. Emerg Infect Dis. 2008;14:1601–9. 18826825 10.3201/eid1410.071042 PMC2609882

[32] Kinsella K, He W. An aging world: 2008. Washington, DC: US Department of Health and Human Services/US Census Bureau; 2009 [cited 2010 Aug 4].

[33] Grills NJ, Rowe SL, Gregory JE, Lester RA, Fielding JE. Evaluation of *Campylobacter* infection surveillance in Victoria. Commun Dis Intell. 2010;34:110–5. 20677420 10.33321/cdi.2010.34.15

[34] Kennedy M, Villar R, Vugia DJ, Rabatsky-Ehr T, Farley MM, Pass M, Hospitalizations and deaths due to *Salmonella* infections, FoodNet, 1996–1999. Clin Infect Dis. 2004;38(Suppl 3):S142–8. 10.1086/381580 15095183

[35] Varma JK, Samuel MC, Marcus R, Hoekstra RM, Medus C, Segler S, *Listeria monocytogenes* infection from foods prepared in a commercial establishment: a case–control study of potential sources of sporadic illness in the United States. Clin Infect Dis. 2007;44:521–8. 10.1086/509920 17243054

[36] Gradel KO, Schonheyder HC, Dethlefsen C, Kristensen B, Ejlertsen T, Nielsen H. Morbidity and mortality of elderly patients with zoonotic *Salmonella* and *Campylobacter*: a population-based study. J Infect. 2008;57:214–22. 10.1016/j.jinf.2008.06.013 18656265

[37] Helms M, Vastrup P, Gerner-Smidt P, Molbak K. Short and long term mortality associated with foodborne bacterial gastrointestinal infections: registry based study. BMJ. 2003;326:357. 10.1136/bmj.326.7385.357 12586666 PMC148890

