# Supplementary material for: Foodborne and Waterborne Infections in Elderly Community and Long-Term Care Facility Residents, Victoria, Australia
Source: Emerg Infect Dis. 2012 Mar;18(3):377–84. doi: 10.3201/eid1803.110311 (PMC3309568; doi:10.3201/eid1803.110311)
Supplement: Technical Appendix — Coding Surveillance Data for Residential Status Surveillance in Victoria, Australia. [file 11-0311-Techapp_3p.pdf]

Article DOI: http://dx.doi.org/10.3201/eid1803.110311

# Foodborne and Waterborne Infections in Elderly Community and Long-Term Care Facility Residents, Victoria, Australia

## Technical Appendix

## Coding Surveillance Data for Residential Status

### Surveillance in Victoria, Australia

In Victoria, Australia, doctors and laboratories were required to notify cases of 64 different infections of public health concern under the Public Health and Wellbeing Regulations 2009 (www.health.vic.gov.au/ideas/notifying/whatto) to the Department of Health, Victoria. Doctors reported cases on handwritten forms or entered details about infected patients online (see www.health.vic.gov.au/notifying). Information provided by doctors included: case-patient's name, date of birth, sex, ethnicity, residential address, infecting agent, date illness began, clinical comments, and contact details for the notifying doctor. Treating doctors were required to sign the notification form. Doctors were able to send, fax, or enter details online or report cases over the phone. Pathology laboratories provided copies of reports to treating doctors about patients infected with diseases on the notification list to the Department of Health. These reports included the patient's name, date of birth, sex, residential address, infecting agent, date of specimen collection, and date a test result was positive.

Department of Health staff members entered details about notified cases onto a database—the Notifiable Infectious Diseases Surveillance System. Where there were discrepancies on duplicate reports from doctors and laboratories, information from the treating doctor's report were entered onto the database. Infections were prioritized for follow-up by public health officers, with more serious or transmissible infections classified as urgent. On the basis of this, public health staff entered additional information on the surveillance database, such as exposure information, vaccination status, any treatment or prophylaxis, and whether the case was part of an outbreak.

## Recording of Addresses

The surveillance dataset recorded address details for cases across 9 fields, that were, in order:

1. Address prefix—for recording information before street number, such as “unit 10.”

2. Street number—recording the street number of the address.

3. Address suffix—records additional information to the street number.

4. Street name—street where the case-patient lived.

5. Address 2—additional information about the address.

6. Address 3—additional information about the address.

7. Suburb—city, town, or suburb where the case-patient lived

8. State—records the case-patients’ residence by state or territory and whether the case-patient was an overseas resident.

9. Postcode—postal code corresponding to the address.

## Assessing Exposure

To assess exposure, 1 investigator (M.D.K.) assessed each address field for all records for names matching that of government-subsidized LTCFs. Names of government-subsidized facilities were obtained from a list held by the Victorian Department of Health in 2010. Where there was doubt about whether a facility named in a case-patient’s record address was an LTCF, the name was checked using a publicly available online aged care guide (www.agedcareguide.com.au/) and the name searched using Google and location searched on Google maps. If any of the following rules were satisfied, a case-patient was coded as living in an LTCF:

1. If the name of a nursing home was included in any of the first 6 address fields and the suburb was recorded as having an LTCF of the same name on the LTCF list or online aged care guide or

2. If the case-patient’s address matched exactly, in terms of street number and street name, that of a recognized government-subsidized LTCF or

3. If the case-patient’s address did not specify a street number but listed a street name where the LTCF list and Internet aged care guide included a facility at that street without a street number recorded or

4. If a Web search on the facility identified that there had been an LTCF of the same name and address at the time of the case-patient's infection but was not currently in operation in 2010.

If a typical name of facilities of any type was mentioned in the first 6 fields and the name was not identified in the LTCF facility lists, a Google search and Google map search were conducted to identify whether the name referred to a retirement village or Supported Residential Service. In instances where the case-patient's address mentioned a "retirement village" but was the same as a recognized LTCF, we searched Google to determine whether there was a retirement village onsite at the same address. If so, the case-patient was coded as living at a "retirement village." All case-patients living in "retirement villages" were considered "community residents" for the purposes of this study.

## Exclusions

In this study, case-patients were excluded if they were not part of the study population according to the following criteria.

1. Nonresidents of Victoria or Australia according to the residential address or country of residence were excluded from the study.

2. Case-patients for which information in any of the first 6 address fields was insufficient to assess whether they lived in an LTCF or the community were not included. Case-patients with a residential address listed as "RMB" (Rural Mail Box) followed by a number were coded as having a valid address. RMB indicated that a person received mail in a rural area at a designated mail box where defined street numbers were not in use. Case-patients with a residential address of PO Box (Post Office Box) followed by a number were coded as missing a valid residential address because it gave no indication of where a case-patient lived.

3. Resident of a "supported residential service"—privately run facilities providing care for people who were old, disabled, or suffering from dementia or psychiatric illness (see www.health.vic.gov.au/srs/index.htm) were not included in the study.
